# Quasi-phase-matched third harmonic generation in organic multilayers

**DOI:** 10.1038/s41598-018-34788-8

**Published:** 2018-11-06

**Authors:** Jiung Kim, CH. S. S. Pavan Kumar, Myoungsik Cha, Heejoo Choi, Kyung-Jo Kim, N. Peyghambarian

**Affiliations:** 1 Department of Physics, Pusan National University, Busan, 609-735 Korea; 2 College of Optical Sciences, University of Arizona, Tucson, AZ 85721 United States

## Abstract

We report the first realization of quasi-phase-matched (QPM) third harmonic generation in isotropic polymer films. Spin-coated thin films of ethyl-violet molecules dispersed in a polymer host (EV) were used as cubic nonlinear optical media because of their transparency at both the fundamental (1230 nm) and the third harmonic (410 nm) wavelengths. A passive layer of a UV-curable material was formed to compensate the phase shift between the two light waves after propagating through each EV layer. We fabricated a series of samples with 1~4 EV layers (0~3 alternatingly coated passive layers). The third harmonic output power showed a quadratic increase with the number of layers, providing a strong evidence for successful quasi-phase-matching. A conversion efficiency of 0.15% was observed with a 190 fs pulse input.

## Introduction

Quasi-phase-matching (QPM) has been widely used in nonlinear optics (NLO) for enhancing quadratic frequency conversion processes such as second harmonic generation (SHG) and parametric down-conversion. QPM in quadratic NLO was efficiently realized with ferroelectric crystals such as LiNbO_3_^1^ and KTiOPO_4_^2^ by periodically reversing the signs of nonlinear optical coefficients in order to compensate the phase shifts between the interacting optical waves during propagation. Efficient third harmonic generation (THG) is also possible in the quadratic NLO by using two successive QPM crystals or a single medium with a properly designed mixture of two QPM periods^3^. In both cases, THG is obtained through SHG, followed by its frequency mixing with the undepleted fundamental (2*ω* + *ω* → 3*ω*).

On the other hand, direct THG is possible in isotropic media where SHG is not involved at all. In particular, polymer films containing some organic molecules are known to have large cubic susceptibilities χ^(3)^, and expected to exhibit efficient THG. Strong green THG was obtained from a polymer film containing nonlinear chromophores and utilized in some applications^4–6^. However, a large *χ*^(3)^ value does not always guarantee a strong THG output. In addition, one needs reasonable transparency at the third harmonic (3*ω*), to avoid its reabsorption. Although the polymer film had a local absorption minimum, it still had a significant loss at 3*ω*, limiting the useful length of the medium to ~5 μm^4^.

Organic molecules with a smaller absorption coefficient in the UV~blue window were studied for efficient THG^7,8^. They are octupolar molecules, crystal violet and ethyl violet, each having a strong resonance near 560 nm^9^, which could result in a large *χ*^(3)^ for THG due partly to the two-photon resonance^10^. Since the 3*ω* (410 nm) light can propagate a relatively long distance without significant loss, and one can think of phase matching or QPM to constructively accumulate the 3*ω* light generated by the fundamental beam at each point along the propagation path.

In isotropic polymer films, however, QPM cannot be realized by modulating the sign of *χ*^(3)^ through electric field poling as in the ferroelectric crystals. Instead, periodic index modulation was suggested for optical fibers to compensate the phase mismatch between the fundamental and the third harmonic light waves^11,12^, and a primitive experimental result was reported^13^. Such a QPM idea has been realized also for high harmonic generation with gas media filled in a modulated hollow core waveguide^14^. However, realization of *χ*^(3)^ QPM in isotropic solid materials has not been reported to our knowledge. Here, we present the first experimental demonstration of QPM THG in isotropic polymer films with alternatingly deposited multilayers of a highly nonlinear organic material and a passive polymer material. The phase mismatch created in the highly nonlinear medium (with large *χ*^(3)^) was compensated in the passive medium (with small *χ*^(3)^). With a proper design and fabrication of the multilayer structure, we could obtain a strong THG output from a multi-layer sample with four periods.

## Design and Theory

**Figure 1:**
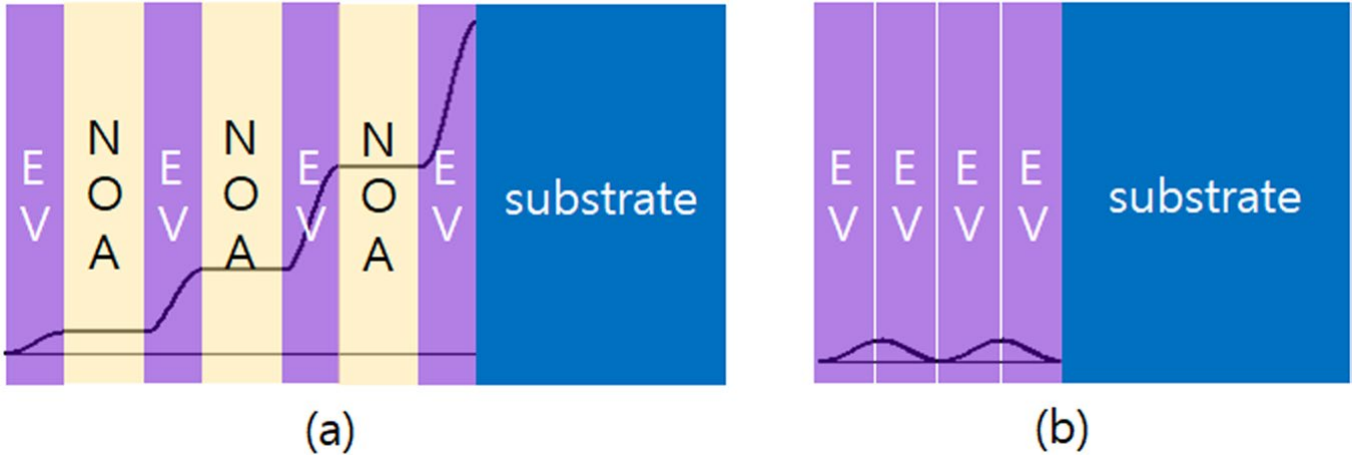
(**a**) A QPM polymer multilayer design with four EV layers (*N* = 4) and three phase-compensating NOA61 layers; the 3*ω* intensity grows during propagation from left to right (solid curve). (**b**) Comparison with a single EV layer with the same total EV-thickness showing no growth of THG. Each layer thickness is equal to the coherence length of the corresponding medium.

A schematic diagram describing our QPM design is given in Fig. 1, showing alternating layers of two materials; an ethyl-cellulose polymer layer containing ethyl-violet molecules (EV) and a UV-curing optical polymer layer (NOA61, Norland Products^15^). EV has a large *χ*^(3)^ value^7^, generating a 3*ω* light, while NOA61 (NOA in short) has a much smaller nonlinearity, which can be treated as a passive phase-compensating layer.

The thickness of each layer was chosen to be one coherence length (*L*_*c*_) for the THG process of upconverting the fundamental at 1230 nm (*ω*) to the third harmonic at 410 nm (3*ω*); *L*_*c*_ = *π*/|Δ*k*|, where Δ*k* = *k*_3*ω*_ − 3*k*_*ω*_ is the wavevector mismatch between them in one-dimensional propagation^10^. This leads to a more practical expression,

1 $${L}_{c}=\frac{\lambda }{6|{n}_{3\omega }-{n}_{\omega }|},$$

where *λ* is the wavelength of the fundamental, and *n*_*ω*_ and *n*_3*ω*_ are the indices of refraction at *ω* and 3*ω*, respectively.

The third harmonic electric field amplitude *E*_3*ω*_ can be obtained from the following one-dimensional wave equation with a nonlinear polarization term proportional to *χ*^(3)^ and the cube of the fundamental electric field;

2 $$\frac{d{E}_{3\omega }}{dz}+\frac{{\alpha }_{3\omega }}{2}{E}_{3\omega }=i\Gamma {{\rm{e}}}^{-i\Delta kz},$$

$${\rm{where}}\,{\Gamma }=\frac{3\pi {\chi }^{(3)}}{4\lambda {n}_{3\omega }}{E}_{\omega }^{3}.$$

Here, we considered the loss (due to absorption or scattering) of the generated third harmonic by introducing a small loss coefficient *a*_3*ω*_, while the loss at the fundamental was neglected.

In the low conversion limit where the fundamental depletion is neglected, the fundamental amplitude *E*_*ω*_ can be treated as a constant, and Eq. (2) can be solved to give an analytic expression of the third harmonic output field amplitude after propagating a distance *z*,

3 $${A}_{3\omega }(z)=U(z){{\rm{e}}}^{-{\alpha }_{3\omega }z/2},$$

4 $${\rm{where}}\,U(z)={A}_{3\omega }(0)+\,\frac{{\Gamma }}{\Delta k+i{\alpha }_{3\omega }/2}[1-{e}^{(\frac{{\alpha }_{3\omega }}{2}-i\Delta k)z}]$$

*A*_3*ω*_(0) is the initial 3*ω* amplitude, which is zero for the first EV layer. When ${\alpha }_{3\omega }/2\ll |\Delta k|$ as in our experimental situation, the second term in Eq. (4) approaches ~*ΓL*_c_ sin(Δ*kz*/2), where one can clearly view how the coherence length defined in Eq. (1) works. When *z* reaches one coherence length (*z* = *L*_*c*_.), the 3*ω* amplitude becomes maximized, and start to decrease with further propagation due to the phase mismatch induced after *L*_*c*_. Therefore, we select the thickness of each EV layer to be *L*_*c*_, and compensate phase shift by placing a passive layer (NOA) with a proper thickness following the EV layer. The correct thickness of the phase compensating layer would be the coherence length for this material, whose dispersion formula is provided by the vendor^15^. It should be noted that Δ*k* > 0 for the NOA layer, while Δ*k* < 0 for the EV layer because its dispersion is anomalous due to the strong absorption resonance located between *ω* and 3*ω*^7^. As a consequence, the phase shift acquired during propagation in EV layer can be compensated to give a zero phase shift at the end of each pair of EV-NOA layers instead of accumulating a phase shift of 2*πN* after propagating *N* pairs, which can be significant at end of the sample when *N* is large. Therefore, a fundamental input with poor temporal coherence can also be used in this QPM scheme, and would produce THG as efficiently as perfectly coherent input as long as the fundamental line width is within the QPM band.

Under an ideal condition that each layer has the correct thickness and no loss (*α*_3*ω*_ = 0), it can be shown that the 3*ω* output intensity increases quadratically with the number of EV layers as depicted in Fig. 1(a). The loss not only limits the useful length (total thickness) of the sample, but also distorts the coherence length for the ideal QPM THG. In our QPM demonstration of four EV layers (*N* = 4), the loss effect was not significant as will be presented below.

## Experiments

First, an ethyl-cellulose polymer containing 20 wt% of ethyl-violet molecules was spin-coated on a 1 mm-thick fused silica substrate to form a 2.7 μm-thick EV layer (*N* = 1 sample). EV has a strong electronic resonance around 560 nm, and transparent in the spectral range of 700 nm~2200 nm. Its magnitude of *χ*^(3)^ was estimated to be ~100 times that of fused silica in the THG Maker fringe experiment by Ramos-Ortiz *et al*.^7^. A unique feature of this material is its excellent transparency between 405 nm and 420 nm. For spin-coated EV thin films (~3 μm or thinner) with good optical quality, the optical density (OD) at the local minimum of 410 nm was too small to be distinguished from the background or the Fresnel reflection losses. Therefore, we estimated only the upper bound of the loss coefficient, *a*_3*ω*_ < 0.03 μm^−1^, by measuring the OD of ~10 μm thick films where scattering was more significant than in thinner films. By the same method, the loss coefficient of NOA was estimated to be ~0.005 μm^−1^, which was much smaller than that of EV, and neglected in our analysis.

The film thickness was chosen to be one coherence length (2.7 μm) for THG of 1230 nm. To determine the coherence length using Eq. (1), one needs accurate values of the index difference at *ω* and 3*ω*. We estimated *n*_*ω*_ = 1.5355 and *n*_3*ω*_ = 1.4585 by transforming the absorption spectrum of a 0.7 μm thick EV film using the Kramers-Kroing relation, which were confirmed by independent index estimations such as critical angle measurement and waveguide coupling method (Metricon, Model 2010 Prism Coupler) at the wavelengths close to *ω* and 3*ω*. Note that *Δk* < 0 (or *n*_3_*ω* < *n*_*ω*_) due to the strong resonance between *ω* and 3*ω*, as we indicated before.

After spin-coating of each EV layer, the residual solvent was allowed to evaporate thoroughly to form a phase compensating layer on it. A UV-curing polymer, NOA61 was spin coated to produce a solid layer with a thickness of one coherence length for this material (5.7 μm), which was determined by Eq. (1) by using *n*_*ω*_ = 1.543 and *n*_3*ω*_ = 1.579. These values were obtained from the Cauchy’s formula for the UV-cured NOA61 film provided by the vendor^15^, which were confirmed by independent index measurement methods as in the EV films. After the coated NOA film was exposed to UV light for curing, the next EV layer was coated on it, completing the *N* = 2 sample. The above procedures were repeated to make *N* = 3 and 4 samples. Before coating each layer, the surface was treated with an O_2_ plasma to enhance adhesion.

**Figure 2:**
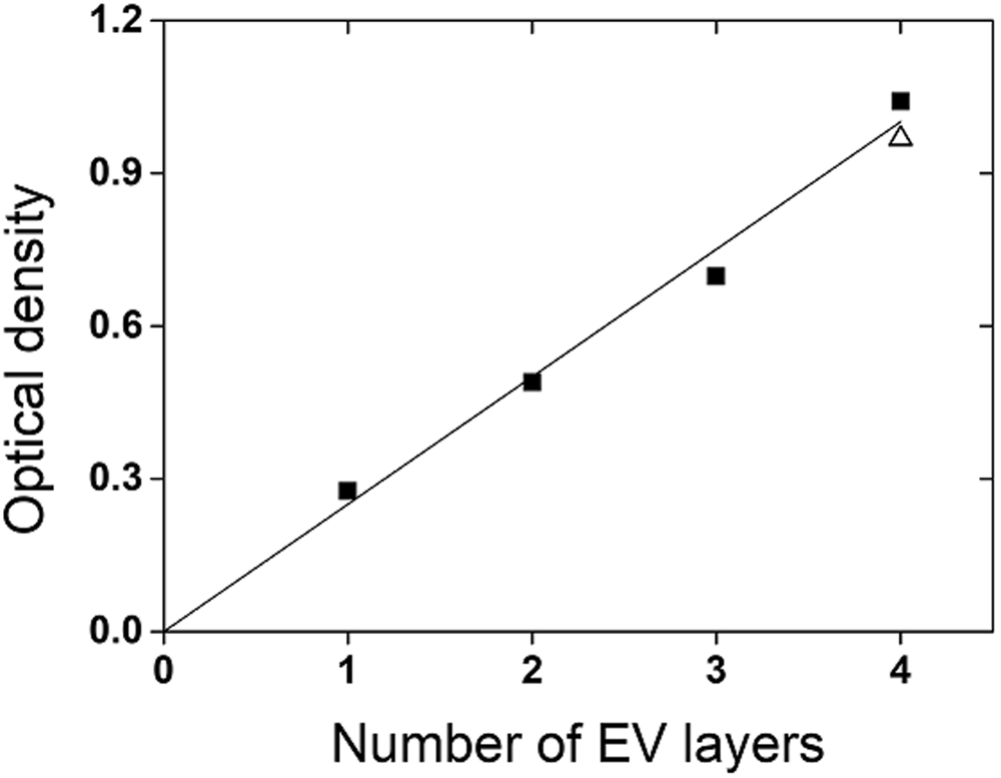
OD at 363 nm versus number of EV layers (1~4). We considered the absorption of the EV layers only, by removing the contribution from the Fresnel reflections at the interfaces. Open triangular symbol indicates the OD of a single EV layer with a thickness of 9.7 μm, which is nearly equal to the total EV thickness of N = 4 sample.

Although we could not verify the detailed structures of the multilayer samples, we estimated the total EV thickness for the samples of *N* = 1~4, by measuring the OD at the local maximum of 363 nm. (OD at the other maxima in the UV~NIR range saturated for thick samples.) The total OD exhibited a linear dependence on the number of layers as shown in Fig. 2, which implies that the EV layers were coated approximately with the designed thickness.

As a light source, we used an optical parametric generation-amplification (OPGA) system employing two β-barium borate crystals, pumped by the third harmonic (355 nm) of a mode-locked Nd:YAG laser (Quantel YG801, pulse width 35 ps.). The idler wavelength of the OPGA was fixed at 1230 nm, aiming at THG at 410 nm. The idler light was loosely focused with a lens on the samples near normal incidence. The 3*ω* output was spectrally separated from the strong fundamental, and measured with a Si-avalanche photodiode. We could not observe any optical damage for the maximum input pulse energy of 100 μJ/pulse, showing a cubic dependence of the 3*ω* output energy on the input pulse energy.

We also used an OPGA pumped Ti:sapphire amplifier (Spectra-Physics, Tsunami, repetition rate 1 kHz, pulse width 190 fs), to get as large THG output as possible. The OPGA was tuned to 1230 nm, and the THG output power was measured with a power meter.

## Results and Discussion

**Figure 3:**
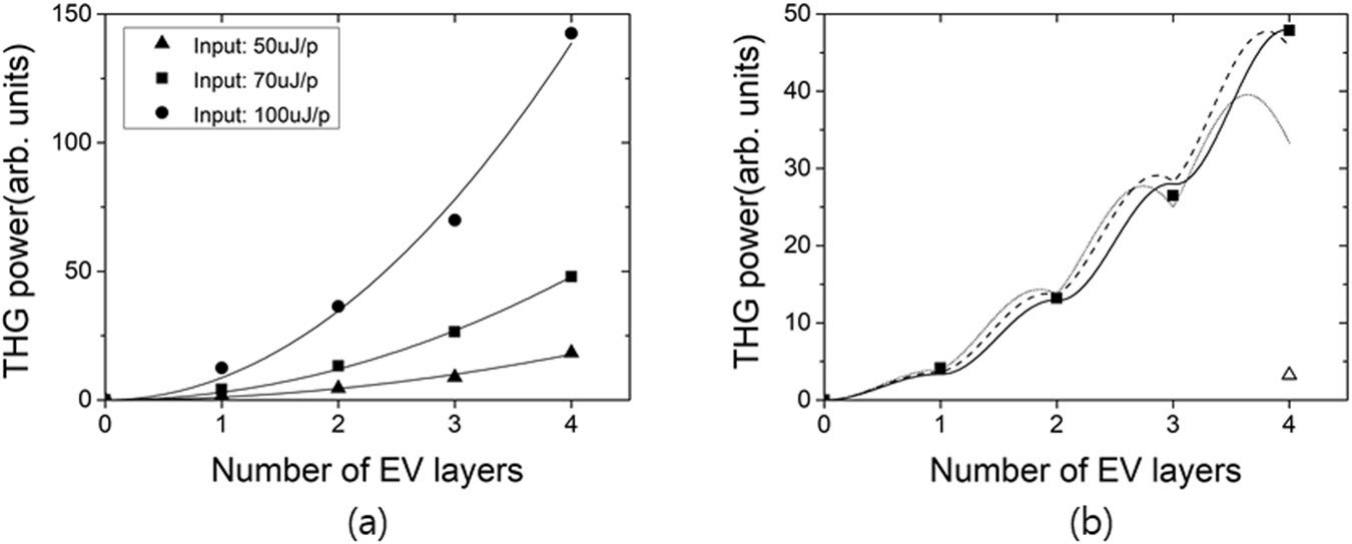
(**a**) THG output power versus number of EV-layers for three different input pulse energies with quadratic fits. (**b**) THG output for input energy of 70 μJ/pulse(middle data in (**a**)) compared with simulation by Eqs (3, 4) (solid curve). Open triangular symbol: THG output power from a single EV layer with a 9.7 μm thickness, which is similar to the total EV thickness *N* = 4 sample. Dashed and dotted curves were calculated THG powers by Eqs (3, 4), assuming 10% and 20% errors in the NOA thickness, respectively, relative to the coherence length.

Figure 3(a) shows the THG output power versus EV layer number *N* for three different input pulse energies at 1230 nm. In each case, the THG output power exhibits quadratic increase with the number of layers (or the propagation length in EV), which can be expected when quasi-phase-matching functions properly. A single layer EV film whose thickness is similar to the total EV thickness for *N* = 4 sample (~10 μm) produced less THG output than the single layer sample (2.7 μm thick), as shown in Fig. 3(b). This clearly demonstrates that our phase compensation scheme was working.

In the calculated 3*ω* intensity in Fig. 3(b), we presented its behavior in the EV layers only, omitting the propagation in the NOA layer, because we assumed that the *χ*^(3)^ of NOA is much smaller than that of EV. Typically, passive transparent polymers such as PMMA are known to have very small *χ*^(3)^ values comparable with that of fused silica^16^, and we assumed that NOA would also have similar nonlinearity. If the *χ*^(3)^ of NOA is comparable with that of EV, THG would be enhanced or reduced depending on the phase difference between the *χ*^(3)^ values of the two media.

Although the quadratic fit in Fig. 3(a) seems to be quite satisfactory, we need to consider the effect of the small loss at 3*ω*. For *a*_3*ω*_ = 0.03 μm^−1^, a 15% decrease in the 3*ω* output intensity was expected after the 4-th EV layer from Eqs (3, 4). In this case, the 3*ω* intensity exhibited a slightly sub-quadratic growth with *N* (approximately *N*^1.9^ dependence for *N* = 4). However, this could not be distinguished clearly from the quadratic dependence in fitting the experimental data.

QPM performance can be hampered by an inaccurate design of each layer thickness (coherence length) or deviation of the layer thickness from the designed value in fabrication. We simulated the behavior of the 3*ω* intensity along the propagation with such thickness errors as follows. First, if we assume that the actual thickness of each layer is ±20% of the corresponding coherence length, the final output after the fourth EV layer was decreased by 9.6% while the (nearly) quadratic growth of the 3*ω* intensity with *N* was maintained, which can be explained by the difference in signs of the phase mismatches in EV and NOA layers, giving a perfect phase compensation.

On the other hand, when there is a thickness error in the NOA layers only (EV layers have the correct thickness), the phase compensation error accumulates along the propagation, and the 3*ω* intensity growth deviates from the ideal quadratic dependence. When there is a ±10% error in the thickness of each NOA layer, it still cannot be clearly distinguished from the ideal quadratic dependence in fitting the experimental data, as shown in Fig. 3(b), dashed curve. However, when a thickness error of ±20% was assumed, we could recognize a significant departure (dotted curve). These simulations imply that the actual thickness errors (uncertainty in the coherence length evaluation or fabrication error) were well within ±20% in our QPM design and experiment. One should note that such thickness tolerance gets tighter when more layers are involved (longer samples).

**Figure 4:**
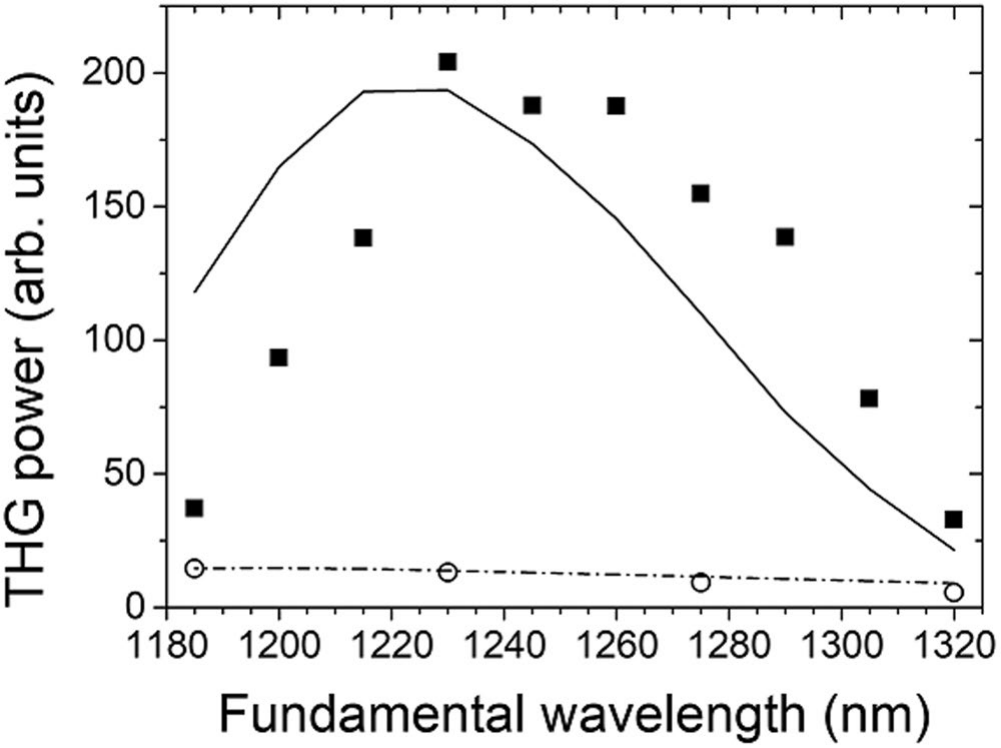
THG output power versus fundamental wavelength for *N* = 1 (open circles) and *N* = 4 (closed squares) samples, compared with theoretical predictions using Eq. (4) (dash-dot and solid lines). The fundamental input was fixed at 70 μJ in the experiments.

For a clearer verification that QPM occurs at 1230 nm, we carried out wavelength-tuned THG experiments. We measured the THG output powers from the *N* = 1 and 4 samples, with the idler of the OPGA tuned at several different wavelengths around 1230 nm, while keeping the power constant. The experimental data in Fig. 4 clearly reveal that QPM occurs around 1230 nm for the *N* = 4 sample. The THG output for *λ* < 1185 nm and *λ* > 1320 nm was too small to be detected. However, although the absorption at 3*ω* limits the useful THG window, our simulation with Eq. (4) revealed that the wavevector mismatch (*Δk*) dominates the QPM behavior for the *N* = 4 sample in the spectral range from *λ* = 1185 nm to 1320 nm. When the wavelength increases, the absolute value of *Δk* for EV increases while *Δk* for NOA decreases, destroying the QPM condition significantly due to the difference in the signs of *Δk* in the two meida.

From the spectra in Fig. 4, we also note that the THG power for the *N* = 1 sample does not vary as significantly as that for *N* = 4. As a result, the power enhancement due to QPM decreases as the wavelength walks away from the QPM wavelength of *λ* = 1230 nm where the power ratio *P*_3*ω*_(*N* = 4)/*P*_*ω*_(*N* = 1) takes a maximum value close to 16.

The discrepancy between the experimental and theoretical spectra for *N* = 4 in Fig. 4 can be attributed to (i) the imperfections in the dispersion formulas for the complex refractive indices for the two materials, (ii) the thickness errors of the layers during fabrication, and (iii) lack of an accurate dispersion information on the *χ*^(3)^. In particular, our theoretical prediction for the spectral range of *λ*_3*ω*_ < 410 nm must have been overestimated because NOA starts absorbing light in this range^15^, which we did not consider in the simulation.

**Figure 5:**
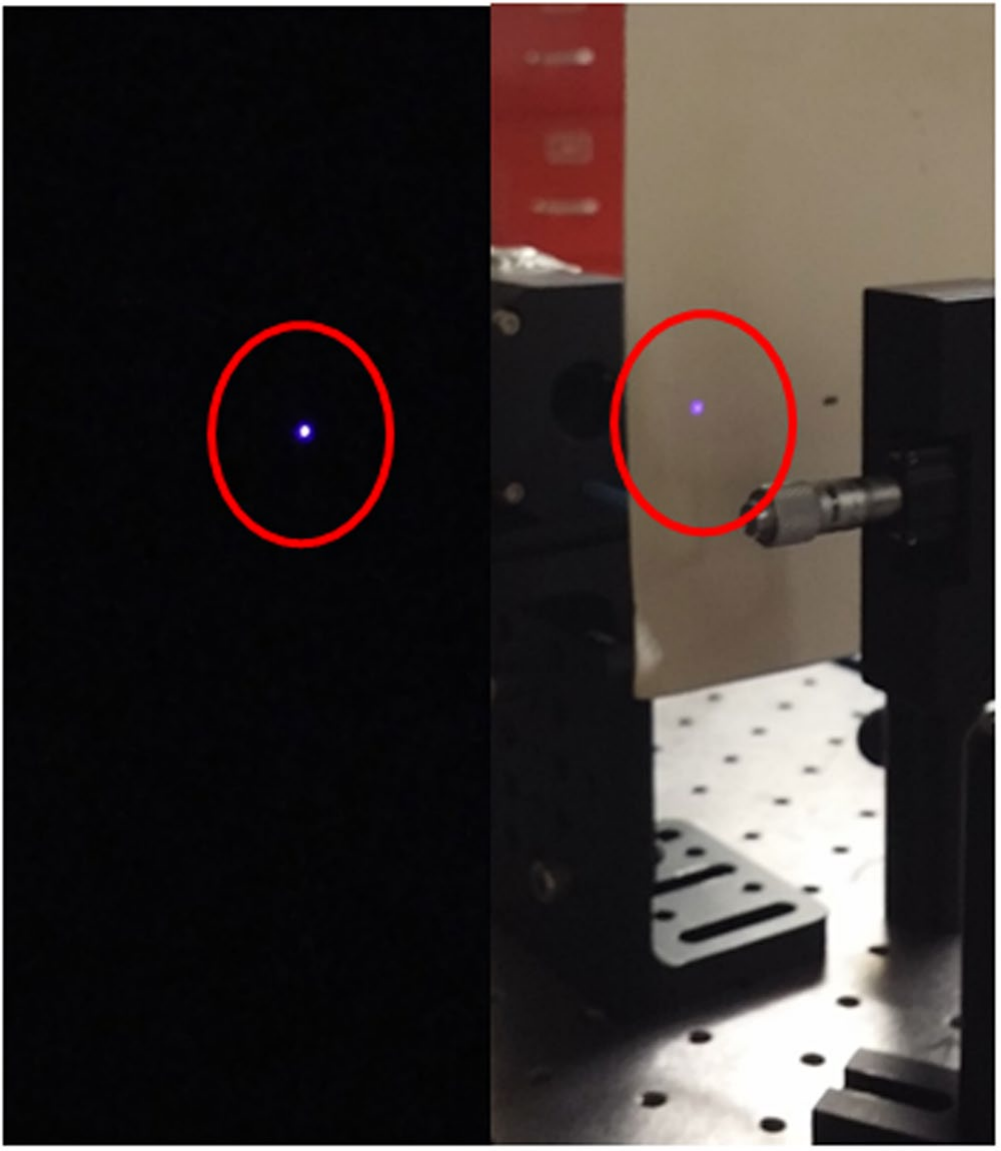
Photographs of violet THG output spots from *N* = 2 sample for a 190 fs pulse input at 1230 nm (30 mW, 1 kHz) in the dark (left) and under room-light (right). (Photographed by two of the authors, HC and KJK).

Finally, the samples were illuminated by 190 fs pulses centered at 1230 nm from the Ti:sapphire-pumped OPGA. When a 30 mW input beam, 1 kHz, was loosely focused on the samples, a bright violet output beams could be viewed with the naked eye, as shown in Fig. 5.

A maximum THG output power of 23 μW was measured for the single layer sample, while one can predict an output power of ~1 mW with the known *χ*^(3)^ value^7^. This large discrepancy may be attributed to the optical damages occurring at this high intensity level. As soon as the input beam started to illuminate, the THG output decreased quickly, saturating to a much smaller value than the one at the very beginning. For the double layer sample (*N* = 2), the output was 46 μW, but did not increase with *N* further, due to the optical damages. Although we used much more intense input than in ref.^4^, this amounts to a record THG conversion efficiency of 0.15%, to our knowledge. The optical damage mechanisms need further investigations to improve the THG efficiency.

## Conclusion

We demonstrated the possibility of QPM for direct THG with multi-layer polymer films. We showed that the THG output increases quadratically with the number of nonlinear layers (or propagation length) up to four periods, and that the QPM condition is met around 410 nm for the third harmonic. Stacking more layers deteriorated the optical quality; the sample tends to lose uniformity quickly and the loss increases significantly. For QPM with more periods, waveguide structure can provide a solution, where thin films with higher optical quality can be properly arranged for QPM with much more degrees of design freedom than the stacked layer structure employed in this study. It is also necessary to investigate new materials having smaller optical losses at the harmonic frequency window.
